# Nomogram for predicting overall survival in patients with invasive micropapillary carcinoma after breast-conserving surgery: A population-based analysis

**DOI:** 10.3389/fsurg.2022.1009149

**Published:** 2022-10-21

**Authors:** Yuting Zhao, Shouyu Li, Lutong Yan, Zejian Yang, Na Chai, Pei Qiu, Jian Zhang, Huimin Zhang, Jianjun He, Can Zhou

**Affiliations:** ^1^ Department of Breast Surgery, The First Affiliated Hospital of Xi’an Jiaotong University, Xi’an, China; ^2^ School of Medicine, Xi’an Jiaotong University, Xi’an, China

**Keywords:** invasive micropapillary carcinoma, breast-conserving surgery, SEER, LASSO regression, nomogram

## Abstract

**Background:**

Due to the loss of prediction of overall survival (OS) for patients with invasive micropapillary carcinoma (IMPC) after breast-conserving surgery (BCS), this study aimed to construct a nomogram for predicting OS in IMPC patients after BCS.

**Methods:**

In total, 481 eligible cases staged 0-III IMPC from 2000 to 2016 were retrieved from the SEER database. A nomogram was built based on the variables selected by LASSO regression to predict the 3-year and 5-year probabilities of OS.

**Results:**

A total of 336 patients were randomly assigned to the training cohort and 145 cases in the validation cohort. The LASSO regression revealed that six variables (age at diagnosis, AJCC stage, marital status, ER status, PR status, and chemotherapy) were predictive variables of OS, and then a nomogram model and an easy-to-use online tool were constructed. The C-indices 0.771 in the training cohort and 0.715 in the validation cohort suggested the robustness of the model. The AUC values for 3-year and 5-year OS in the training cohort were 0.782, 0.790, and 0.674, and 0.682 in the validation cohort, respectively. Based on the cutoff values of 147.23 and 222.44 scores calculated by X-tile analysis, participants in the low-risk group (≤147.23 scores) had a more favorable OS in comparison with those in the medium (>147.23, but <222.44 scores)- and high-risk groups (≥222.44 scores).

**Conclusions:**

By risk stratification, this model is expected to provide a precise and personalized prediction of the cumulative risk and guide treatment decision-making in improving OS strategies for IMPC patients.

## Introduction

Invasive micropapillary carcinoma (IMPC), consisting of small, hollow, or morula-like clusters of cancer cells surrounded by clear stromal spaces, is a rare histologic subtype of breast cancer (1–3). Previous researches have shown that IMPC tends to show aggressive clinical characteristics, such as a significant tendency for lymphovascular invasion (LVI), lymph node metastasis (LNM), and poor prognosis (4–6). Consequently, IMPC has always been over-treated with unnecessary chemotherapy in the past 20 years. One of the main reasons is the limited knowledge on IMPC based on clinical literature research owing to the rarity of this breast malignancy (6, 7).

As one of the standard treatments of breast cancer, breast-conserving surgery (BCS) followed by radiotherapy has excellent results on overall survival (OS) and relapse-free survival and has been widely performed in recent years (8, 9). The efficacy of BCS on IDC rather than IMPC has been extensively evaluated. And the precise clinical value of BCS for patients with IMPC is still unknown, due to the lack of large, randomized controlled trials that investigated the prognostic prediction factors of the OS probability for patients with IMPC after BCS. For these reasons, the confirmation of the real-world therapeutic effectiveness of BCS for IMPC patients is urgently needed.

To further explore the therapeutic effect of BCS on IMPC, a large population of female IMPC patients is investigated through the Surveillance, Epidemiology, and End Results (SEER) database (10, 11). A nomogram is established and validated based on prognostic factors from the LASSO regression to obtain predicted survival probabilities in IMPC patients who underwent BCS. This model is expected to provide guidance on the OS probability for IMPC patients with BCS through important prognosis markers and facilitate the decision-making of follow-up treatment for clinicians.

## Materials and methods

### Data source and patient population

**Figure 1:**
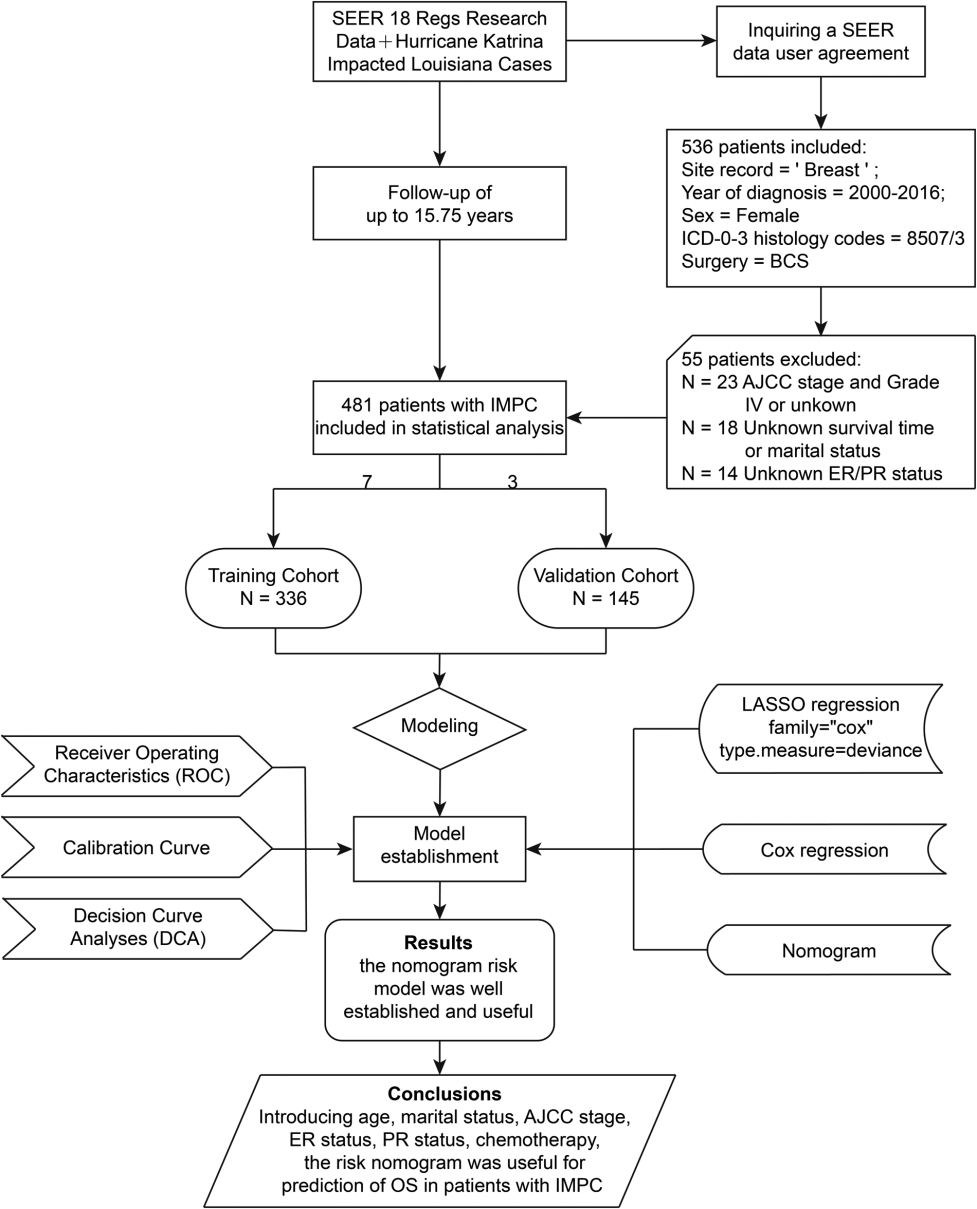
Flowchart of the study design. AJCC, American Joint Committee on Cancer; BCS, breast-conserving surgery; ER, estrogen receptor; IMPC, invasive micropapillary carcinoma; LASSO, least absolute shrinkage and selection operator; OS, overall survival; PR, progesterone receptor; SEER, the Surveillance, Epidemiology, and End Results database.

For the current study, data were derived from SEER 18 Regs Research Data with a data user agreement. The SEER*Stat software program (version 8.3.8, http://seer.cancer.gov/seerstat) (Information Management Service, Inc. Calverton, MD, USA) was administrated to select female patients diagnosed with IMPC undergoing BCS from 2000 to 2016 with the inclusion criteria listed as follows: (1) primary tumor site code 8507/3: Ductal carcinoma, micropapillary in the International Classification of Disease for Oncology, third edition (ICD-O-3); (2) Breast-Adjusted American Joint Committee on Cancer (AJCC) 6th stages 0, I, II or III; (3) cancer-directed surgery recode BCS. The exclusion criteria were listed as follows: (1) unknown age at diagnosis, marital status, histopathological differentiation grade, AJCC stage, survival time, estrogen receptor (ER) status or progesterone receptor (PR) status; (2) the diagnosis methods of autopsy only or death certificate; (3) AJCC stage IV or grade IV; (4) missing surgical records, or surgery not performed. The screening process and study design were presented in Figure 1.

The studied variables included were age at diagnosis, race, years of diagnosis, marital status, histopathological grade, breast-adjusted AJCC 6th TNM stage, ER status, PR status, HER-2 status, radiotherapy, chemotherapy, survival months, vital status, cause of death, type of surgical procedures, and so on. All methods were performed in accordance with the relevant guidelines and regulations. This study was approved by the Ethical Committee of the First Affiliated Hospital of Xi'an Jiaotong University. The SEER data erases the identity information of patients, so there is no need for informed consent from the patients.

### Data collection and endpoint

The median follow-up time of the study cohort was 4.92 years (range from 1 month to 15.75 years). The endpoint of the current study was overall survival (OS), which was defined as the time from the date of diagnosis until death from any cause. All the patients enrolled were effectively followed up, and the final follow-up time: November 2018.

### Statistical analysis

Patients were randomly split to the training cohort and the validation cohort (7:3) by R software *rms* package. Differences between the cohorts were compared by Pearson's chi-square test or Fisher's exact test when needed. In the training cohort, hazard ratios (HRs) with 95% confidence intervals (CIs) were calculated through Cox proportional hazards model to estimate prognosis. The least absolute shrinkage and selection operator (LASSO) regression was performed to screen out predictive variables to generate a nomogram. As a shrinkage estimate, LASSO obtains a more refined model by constructing a penalty function. The penalty function shrinks the regression coefficients of LASSO, that is, the sum of the absolute values of the forced coefficients is less than a fixed value. Meanwhile, some regression coefficients of LASSO are set to zero. Through continuous shrinkage operation, the regression coefficient is minimized to reduce the possibility of over-fitting.

The LASSO regression was performed using the *glmnet* package of R software. The family = “cox” was set to study the relationship between predictor variables and survival time. The function of *cv.glmnet* returned a list with all the ingredients of a ten-fold cross-validated fit to select *λ*. The value of *λ* obtained by lambda.1se gave the most regularized model that the cross-validated error was within one standard error of the minimum. With the selected *λ* value of 0.0481272, six predictor variables including age at diagnosis, AJCC stage, marital status, estrogen receptor (ER) status, progesterone receptor (PR) status, and chemotherapy were screened out and allowed to enter the nomogram model.

A Cox regression model was built in the training cohort with the predictor variables screened out by the LASSO regression. Based on the contributions of the variables to the model, the nomogram was constructed to predict the OS probability using R software *rms* package. Harrell's concordance-index (C-index) and receiver operating characteristics (ROC) curves were performed to evaluate the discriminatory ability of the nomogram. Calibration curves were plotted to evaluate the consistency between the predicted and the observed probability. Decision curve analyses (DCA) were performed for clinical usefulness assessment of this predictive model.

Combined with the coefficient of each variable in the Cox regression model, a scoring system was calculated through the nomogram built in the training cohort (12). And the training cohort was split into high-, medium- and low-risk groups with the cut-off values of the total score determined by the X-tile software, which was employed to determine the cutoff points of the optimal score by comparing the survival and product a minimum *P*-value.

Survival curves by risk level and other significant variables for the study patients were calculated using the Kaplan-Meier analysis and compared across groups by log-rank test. Two-sided *P* < 0.05 was considered to have statistical significance. Statistical analyses were performed by software package R version 4.0.2 and X-tile version 3.6.1.

## Results

### Patient characteristics

A total of 481 participants diagnosed with IMPC were included in the current study, as shown in Table 1. Totally, 336 eligible subjects were randomly assigned to the training cohort and 145 were randomly assigned to the validation cohort. Among the 481 subjects included, the mean age was 58.13 (±14.17) years old, 58.6% (282/481) of them were White race, and 56.5% (272/481) of them were married women. The majority of IMPC cases were moderately and poorly differentiated (grade II and III) (95.20%, 458/481), AJCC stage III (54.50%, 262/481), ER-positive (85.00%, 409/481), PR-positive (72.30%, 348/481) and HER-2 negative (73.90%, 212/287) tumors. In total, 46.8% (225/481) of patients received radiotherapy, and 72.60% (349/481) had undergone chemotherapy. No significant differences in the clinicopathologic characteristics were observed between training and validation cohorts.

**Table 1:** Patient clinical and pathological characteristics.

Characteristics	Total *n* = 481		Training Cohort *n* = 336		Validation Cohort *n* = 145		*χ* ^2^	*P*-value*
Age at diagnosis								
<40	50	10.40%	34	10.10%	16	11.00%	0.146	0.929
40–64	267	55.50%	186	55.40%	81	55.90%		
≥65	164	34.10%	116	34.50%	48	33.10%		
Race								
White	282	58.60%	198	58.90%	84	57.90%	0.264	0.876
Black	70	14.60%	50	14.90%	20	13.80%		
Others	129	26.80%	88	26.20%	41	28.30%		
Year of diagnosis								
2000–2005	72	15.00%	50	14.90%	22	15.20%	0.020	0.990
2006–2011	178	37.00%	125	37.20%	53	36.60%		
2012–2016	231	48.00%	161	47.90%	70	48.30%		
Marital status								
Married	272	56.50%	185	55.10%	87	60.00%	1.006	0.316
Unmarried/Loss of marriage	209	43.50%	151	44.90%	58	40.00%		
Grade								
I	23	4.80%	12	3.60%	11	7.60%	3.863	0.145
II	227	47.20%	158	47.00%	69	47.60%		
III	231	48.00%	166	49.40%	65	44.80%		
AJCC Stage								
0-I	55	11.40%	39	11.60%	16	11.00%	0.557	0.757
II	164	34.10%	111	33.00%	53	36.60%		
III	262	54.50%	186	55.40%	76	52.40%		
ER Status								
Positive	409	85.00%	292	86.90%	117	80.70%	3.074	0.080
Negative	72	15.00%	44	13.10%	28	19.30%		
PR Status								
Positive	348	72.30%	243	72.30%	105	72.40%	<.001	0.983
Negative	133	27.70%	93	27.70%	40	27.60%		
HER2 Status^a^								
Positive	75	26.10%	55	27.40%	20	23.30%	0.526	0.468
Negative	212	73.90%	146	72.60%	66	76.70%		
Radiotherapy								
Yes	225	46.80%	157	46.70%	68	46.90%	0.001	0.973
No	256	53.20%	179	53.30%	77	53.10%		
Chemotherapy								
Yes	349	72.60%	238	70.80%	111	76.60%	1.663	0.197
No	132	27.40%	98	29.20%	34	23.40%		

ER, estrogen receptor; PR, progesterone receptor. HER-2, human epidermal growth factor receptor 2. AJCC, American Joint Committee on Cancer.

* *P*-values showed whether there were statistical differences among, new *P*-values shall be shown, when necessary, in the below.

^a^ The SEER database only recorded HER-2 status after January 1, 2010 (317/707).

### Risk factors for os in the training cohort

To preliminarily estimate survival outcomes, a series of survival analyses were conducted. Among the 481 patients in the current study, a total of 84 (17.5%) patients died during the median follow-up of 4.92 years (range from 1 month to 15.75 years), and 64 (19.4%, 64/336) cases of them were in the training cohort while 20 (13.7%, 20/145) cases in the validation cohort, respectively.

**Figure 2:**
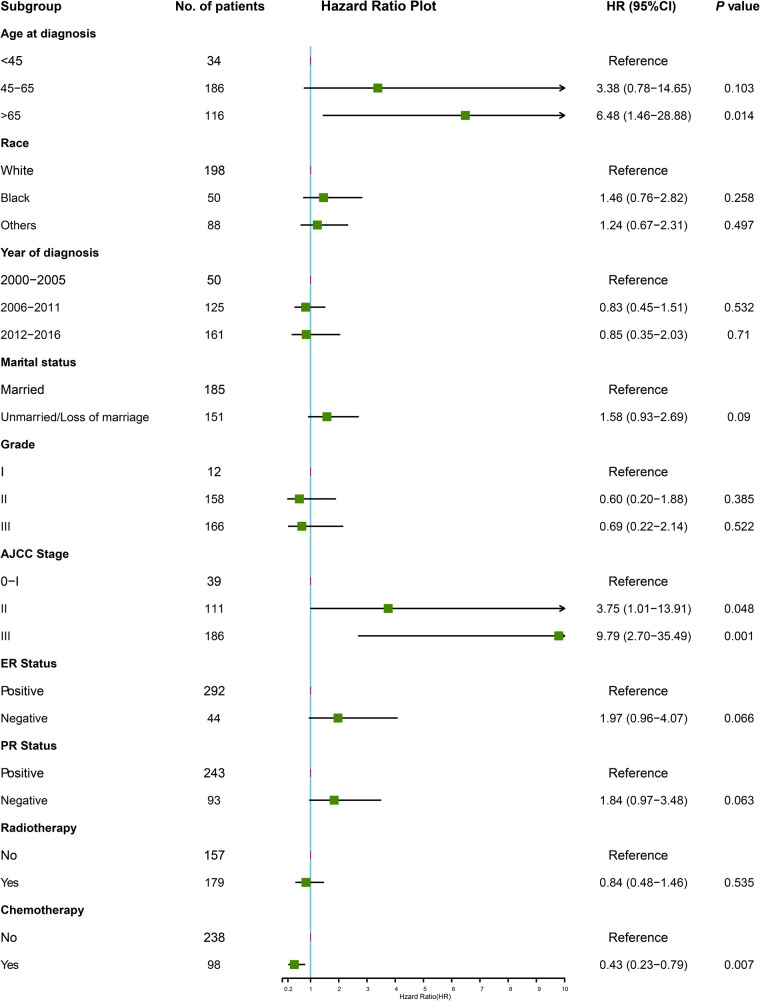
The forest plot for multivariate Cox analysis in the training cohort. AJCC, American Joint Committee on Cancer; ER, estrogen receptor; HR, hazard ratio; PR, progesterone receptor.

Multivariable Cox regression analysis was adopted to identify the independent risk factors affecting OS. In the training cohort, six variables were significantly associated with OS. Adverse prognostic factors included age at diagnosis (65 years and older, HR = 6.48, *P* = 0.014), marital status (unmarried or loss of marriage, HR = 1.58, *P* = 0.090), AJCC stage (stage II, HR = 3.75, *P* = 0.048, stage III, HR = 9.79, *P* = 0.001), ER-negative tumors (HR = 1.97, *P* = 0.066), PR negative tumors (HR = 1.84, *P* = 0.063), and favorable factor only included chemotherapy (HR = 0.43, *P* = 0.007), in terms of OS (Figure 2).

### Independent risk factors in the training set

**Figure 3:**
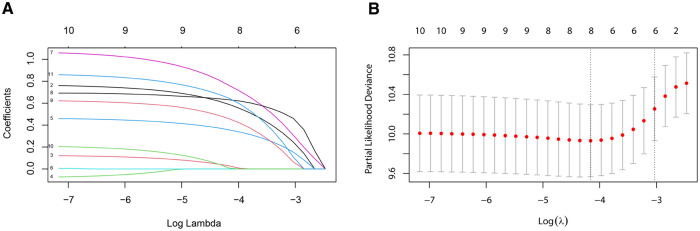
Variable selection by the LASSO binary logistic regression model. A coefficient profile plot was constructed against the log (lambda) sequence. (**A**) Six variables with nonzero coefficients were selected by deriving the optimal lambda. (**B**) Following verification of the optimal parameter (lambda) in the LASSO model, we plotted the partial likelihood deviance curve vs. log (lambda) and drew dotted vertical lines based on 1 standard error criteria. LASSO, least absolute shrinkage and selection operator.

Six predictive variables with nonzero coefficients, including age at diagnosis, marital status, AJCC stage, ER status, PR status, and chemotherapy, were identified by the LASSO regression (Figure 3).

### Predictive model construction

**Figure 4:**
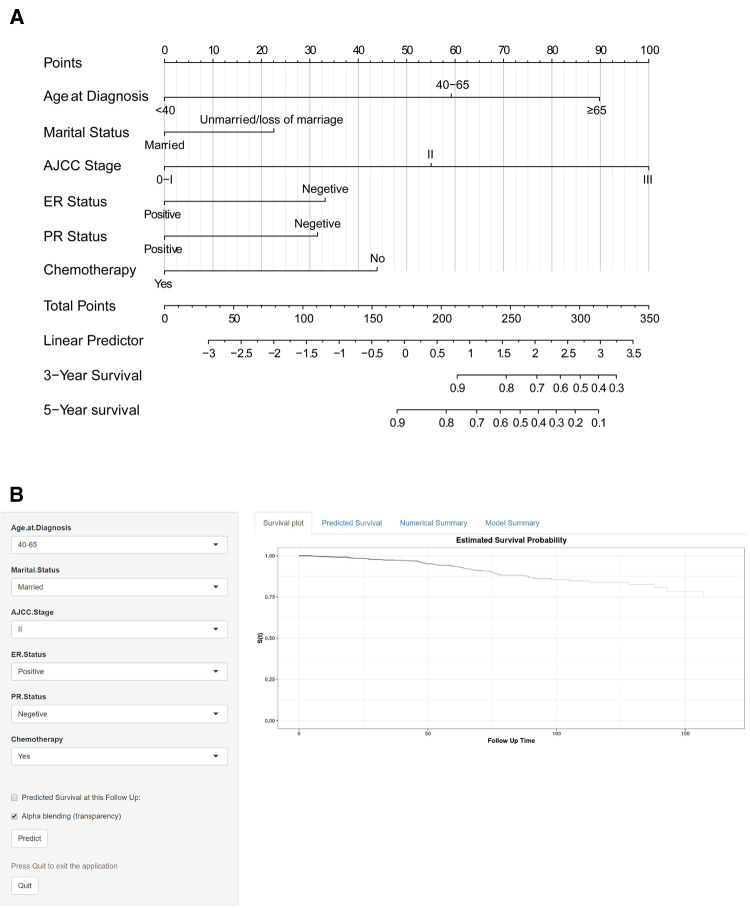
The (**A**) prognostic nomogram and (**B**) the online dynamic nomogram for 3- and 5-year overall survival (OS). When using the nomogram model, the upward vertical line drawn for each variable was adopted to obtain its risk score. Total points were calculated by adding each risk score and drawing a downward vertical line, which illustrated the probability of 3- and 5-year OS. AJCC, American Joint Committee on Cancer; ER, estrogen receptor; PR, progesterone receptor.

A nomogram was constructed based on the predictive variables identified from the LASSO regression and multivariable Cox regression analysis in the training cohort (Figure 4A). The nomogram demonstrated that the AJCC stage had the greatest influence on the prediction of OS, followed by age at diagnosis and chemotherapy. ER status, PR status, and marital status also revealed moderate influences on OS. The 3- and 5-year survival probabilities were easy to calculate by using the nomogram tool. To make sure more intuitive prediction results, an easy-to-use web tool of dynamic nomogram (https://asmallail.shinyapps.io/DynNomapp/) was designed (Figure 4B). The survival probability and survival curve could be predicted by simply entering the information of a patient.

Take an IMPC female patient diagnosed at the age of 60 as an example, married, AJCC stage II, ER positive and PR negative tumor, and treatment with BCS and chemotherapy would score a total point of 145.9, by drawing a downward vertical line or entering the information of the patient online, we could obtain the probabilities of 3- or 5-year survival for this patient were 97.0% and 94.0%, respectively.

### Validation and calibration of the nomogram model

**Figure 5:**
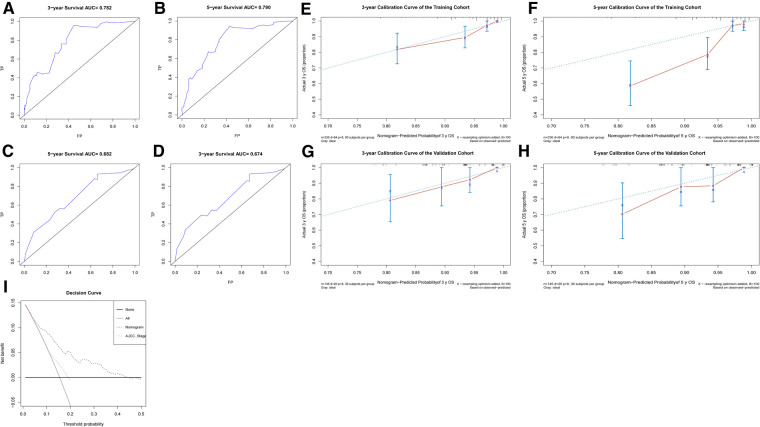
The predictive performance evaluation of this nomogram. (**A–D**) The ROC curves of the training cohort (**A,B**) and the validation cohort (**C,D**); (**E–H**) the calibration curves of the training cohort (**E,F**) and the validation cohort (**G,H**); (**I**) The DCA curves for nomogram and AJCC TNM staging of the total cohort. AJCC, American Joint Committee on Cancer; DCA, decision curve analysis; ROC, receiver operator characteristic.

To access the predictive performance of this model, the discrimination, calibration, and clinical usefulness were assessed both in the training and validation cohorts. C-index was calculated, and ROC curves were plotted to show the discrimination of this nomogram model. The C-indices were 0.771 (95% CI, 0.712–0.830) and 0.715 (95% CI, 0.603–0.827) in the training and validation cohorts, respectively. As shown in Figures 5A–D, area under the curve (AUC) values for 3-year and 5-year OS in the training cohort were 0.782, 0.790, respectively, and 0.674, 0.682 in the validation cohort, respectively. The calibration curves were employed to assess the calibration, which demonstrated good consistency between the predicted and the observed probability (Figures 5E–H). As shown in Figure 5I, decision curve analysis (DCA) curves illustrated that when the threshold ranged from 0.05 to 0.45, the net benefit would be positive by using this nomogram, and exhibited a more favorable net benefit for 3- and 5-year OS prediction and a better potential clinical usefulness of this model, when compared to AJCC TNM staging system.

### The construction of risk stratification

**Figure 6:**
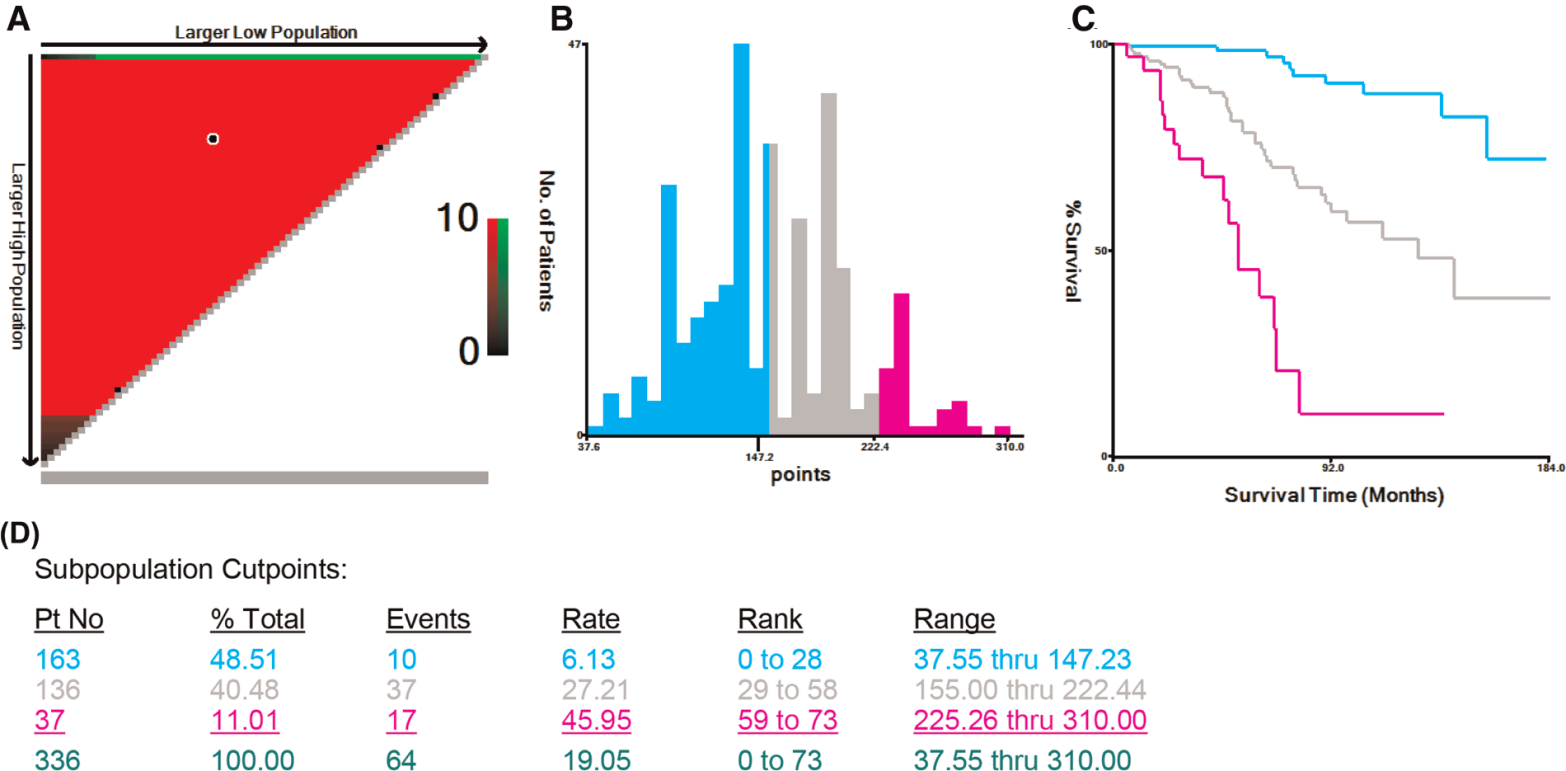
X-tile analysis of survival data from the Surveillance, Epidemiology, and End Results registry, including the X-tile plot (**A**), a histogram (**B**), the Kaplan-Meier curve (**C**), and the data (**D**) related to optimal cut-point.

To further validate the predictive ability of this nomogram, three risk classifications, low-, medium- and high-risk groups, were constructed based on the cutoff values of the calculated total score in the training cohort by X-tile software. With the cutoff values of 147.23 and 222.44 scores determined by X-tile analysis (Figure 6), 48.51% (163/336) eligible subjects were classified into the low-risk group (≤147.23 scores), 40.48% (136/336) were classified into the medium-risk group (>147.23, but ≤222.44 scores), while 11.01% (37/336) ones were classified into the high-risk group (>222.44 scores).

### Kaplan-Meier survival analyses by risk level

**Figure 7:**
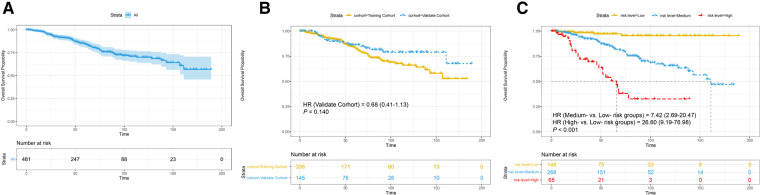
Survival analysis (**A**) in the total cohort. Subgroup analyses stratified (**B**) by cohort; (**C**) by risk level. HR, hazard ratio. *P*-value was calculated by log-rank test.

As shown in Figures 7A, B, the cumulative incidences of OS at 3, 5, and 10 years were 91.60%, 84.50%, and 70.00% in the total cohort, respectively, 92.50%, 83.80%, and 66.10% in the training cohort, respectively, and 89.80%, 86.30%, and 79.00% in the validation cohort, respectively. However, no significant improvement in OS for patients in the validation cohort (HR = 0.68, 95% CI, 0.41–1.13, *P* = 0.140) when compared to patients in the training cohort (Figure 7B).

As shown in Figure 7C, the 10-year cumulative incidences of OS were 95.5% in the low-risk group, 87.4% in the medium-risk group, and 33.9% in the high-risk group. The Kaplan-Meier curves illustrated that participants in the low-risk group had a more favorable OS in comparison with those in the medium- and high-risk groups (medium-risk vs. low-risk, HR = 7.42, 95% CI, 2.69–20.47, high-risk vs. low-risk, HR = 26.60, 95% CI, 9.19–76.98, *P* < 0.001), which demonstrated a strong correlation of the total score calculated by our nomogram analysis and 10-year OS.

## Discussion

In this research, a predictive model was built based on clinicopathologic features in a large cohort with extended follow-up to predict long-term survival in IMPC patients after BCS. The nomogram model showed a good predictive performance for 3- and 5-year OS after strictly calibrating and validating in both training and validation cohorts. So far as we knew, the current study with a large population firstly established a nomogram to provide the predictive basis for OS of IMPC patients after BCS. Analyses of demographic variables and clinicopathological factors could provide guidance on the OS and help the decision-making for clinicians and IMPC patients with BCS.

As reported by previous studies (13–16), the objective and reliable prognostic indicators, such as AJCC stage, age at diagnosis, and ER status, have always been seemed as the clinicopathological features which could direct clinical treatment for breast malignancies. In this study, based on the six selected predictive variables affecting OS for IMPC patients undergoing BCS, the nomogram suggested that the AJCC stage contributed most to prognostic prediction, followed by age at diagnosis, ER status, and PR status. Similar to previous research (17–19), it was also found in this study that prognostic markers, such as age at diagnosis, stage, and hormone receptor status, were long-term survival factors contributing to improved or decreased OS for patients with IMPC. A high tendency of lymph node metastasis was reported in previous IMPC series (20–22), which was closely associated with survival outcomes for breast cancer patients (23). Consequently, it can be inferred that the lymph node (N) stage utilized by the AJCC has a significant influence on the prognosis of IMPC. We also noted that marital status made a moderate contribution to OS, and those who were unmarried or loss of marriage were more at risk in terms of OS. The underlying reasons might be that married patients tended to have more financial resources, the chance of surgery, and psychosocial support (24), which resulted in a more favorable clinical outcome.

The high tendency of lymphatic vascular invasion, lymph node involvement, as well as a high locoregional recurrence (LRR) risk were supposed to be aggressive clinicopathologic features of IMPC in the previous studies (25–28). Moreover, OS has always seemed as an unbiased measurement for survival prediction of breast cancer (13, 14). In the current research, the 5-year OS for conservatively operated IMPC patients was 80.9%, which was slightly lower than 82.9%–90.0% in recent studies (15, 16, 28, 29). Unexpectedly, the corresponding reasons for the favorable overall survival were still unknown (1, 6, 22). Previous studies had identified that a large percentage of hormone receptor-positive tumors might contribute to the relatively good prognosis of IMPC (28, 30, 31). In our study, the ER and PR positivity rates of 85.00% and 72.30%, respectively, were consistent with the reported literature that ranged from 20% to 88% (6, 32–34). In addition, ER-negative tumors were correlated with poor OS (ER-negative vs. ER-positive: HR = 1.97) for IMPC patients with BCS and contributed moderately to the nomogram model in our study. Such findings demonstrated that ER status could be adopted to predict the prognosis probability for IMPC patients after BCS. The underlying reasons could be attributed to endocrine therapy, which could reduce the potential risk of LRR and improve long-term prognosis (31). Furthermore, we noted that younger age at diagnosis was a favorable factor for conservatively operated IMPC patients, which was similar to some previous IMPC series (6, 27, 31). On the one hand, the elderly with chronic diseases probably affected overall survival. On the other hand, advances in the screening of breast cancer and comprehensive treatment could attribute to a better outcome for young IMPC patients. However, due to the lack of prospective studies on IMPC of the breast, the prediction of long-term survival outcomes for IMPC patients after BCS is still required.

According to current guidelines, BCS followed by radiotherapy is one of the standard treatments for invasive breast carcinoma. Correspondingly, IMPC patients were also prone to accept BCS rather than mastectomy in the previous series (5, 17, 26). However, the precise prognosis value of BCS for patients with IMPC remained unknowable. To provide more accurate evidence on prognosis estimation and therapy evaluation in patients with IMPC, a nomogram was constructed based on the predictive factors identified by the LASSO regression in the training cohort. For instance, if an unmarried woman was diagnosed with stage III and HR-positive IMPC when she was 45 years old and had been treated with BCS but without chemotherapy, the OS probabilities at 3- and 5-year were as high as 87.0% and 70.0%, respectively. Therefore, the nomogram in the present study appeared to be helpful to assess the long-term prognosis, develop therapeutic strategies and improve the compliance of IMPC patients.

Adjuvant/neoadjuvant chemotherapy, which is known to reduce the invasiveness and metastasis of breast cancer, is an essential part of comprehensive treatment for breast malignancies and the mainstay of treatment for IMPC patients. After eliminating the confounding factors, the multivariate Cox analyses also revealed chemotherapy as a favorable prognostic factor. Furthermore, in the current nomogram, if the woman patient mentioned above had undergone chemotherapy, the 3- and 5-year cumulative probabilities of survival would be improved to 95.0% and 87.0%, respectively. Consequently, the administration of chemotherapy could improve the probabilities of OS for IMPC patients and prolong survival time. However, further basic and prospective clinical studies are still warranted.

In addition, the predictive performance of the current nomogram was strictly measured on the aspects of discrimination, calibration, and clinical usefulness. The C-indices of 0.771 and 0.715 in training and validation cohorts indicated a good discriminatory power of the model and revealed that approximately 75% of the time this nomogram could help predict the prognosis accurately. In contrast with the AJCC staging system, this nomogram showed more favorable clinical usefulness in decision curve analysis and then provided more valuable information for clinical decision-making through incorporating more clinicopathological parameters. A risk score system was also established to validate the ability of the model to discriminate between patients at different risk levels. If the total scores of patients were higher than the cut-off value of 222.44, the patients would be identified with poorer clinical outcomes and needed further evaluation and more improved comprehensive therapeutic strategies. Consequently, the current nomogram could provide a more accurate prognostic prediction for individual IMPC patients after BCS and be applied to make clinical treatment strategies. Meanwhile, taking into account of the complexity of score calculation and inconvenient application in clinical practice, the visual nomogram and the online tool could be applied to calculate the OS probabilities and gave clinicians a more accurate assessment of the prognosis for IMPC patients after BCS.

Reflecting on the limitations of this study and the inherent disadvantages of using the SEER database (14), some conclusions must be interpreted with caution. Firstly, the design of the study was retrospective and inevitably suffered from possible biases, such as Neyman's bias, Berkson's bias, and lead-time bias. Secondly, some essential information was absent in our study and could not be analyzed, such as information on family history, insurance, comorbidities, the percentage of IMPC component, HER-2 status, as well as chemotherapy regimens. Thirdly, records on recurrence and failure patterns were not provided in this database and could not be investigated. Fourthly, statistical significance was defined by *P* < 0.05 without adjustment in multiple analyses, consequently the type I error might exceed 0.05. Moreover, although the nomogram was constructed based on a large population and validated both in the training and validation cohorts, it still needed to be externally validated in other populations.

## Conclusions

In summary, this study found that older age at diagnosis, the inexistence of marriage status, late AJCC stage, ER-negative or PR-negative tumors, and not receiving chemotherapy were independent risk factors affecting OS for IMPC patients after BCS. Based on the identified variables selected by the LASSO regression to provide a prognostic prediction for conservatively operated IMPC patients, we developed and validated a nomogram that could accurately predict the individualized risk of OS for patients with IMPC after BCS. By identifying the score for each patient, this model was expected to offer guidance for making treatment decisions in improving long-term follow-up strategies. Randomized controlled clinical trials with long follow-up times are still needed to provide a high level of evidence on the predictive probabilities of OS for conservatively operated IMPC patients.

## Data Availability

'The original contributions presented in the study are included in the article/Supplementary Materials, further inquiries can be directed to the corresponding author/s.
